# Circular Economy of Construction and Demolition Waste: A Literature Review on Lessons, Challenges, and Benefits

**DOI:** 10.3390/ma15010076

**Published:** 2021-12-23

**Authors:** Callun Keith Purchase, Dhafer Manna Al Zulayq, Bio Talakatoa O’Brien, Matthew Joseph Kowalewski, Aydin Berenjian, Amir Hossein Tarighaleslami, Mostafa Seifan

**Affiliations:** School of Engineering, The University of Waikato, Hamilton 3240, New Zealand; ckp8@students.waikato.ac.nz (C.K.P.); dmsa2@students.waikato.ac.nz (D.M.A.Z.); bo22@students.waikato.ac.nz (B.T.O.); mjk38@students.waikato.ac.nz (M.J.K.); aydin.berenjian@waikato.ac.nz (A.B.); amir.tarighaleslami@waikato.ac.nz (A.H.T.)

**Keywords:** construction and demolition waste, circular economy, 3R principle, waste management, construction, CO_2_ emission, recycling

## Abstract

Conventionally, in a linear economy, C&D (Construction and Demolition) waste was considered as zero value materials, and, as a result of that, most C&D waste materials ended up in landfills. In recent years, with the increase in the awareness around sustainability and resource management, various countries have started to explore new models to minimize the use of limited resources which are currently overused, mismanaged, or quickly depleting. In this regard, the implementation of CE (Circular Economy) has emerged as a potential model to minimize the negative impact of C&D wastes on the environment. However, there are some challenges hindering a full transition to CE in the construction and demolition sectors. Therefore, this review paper aims to critically scrutinize different aspects of C&D waste and how CE can be integrated into construction projects. Reviewing of the literature revealed that the barriers in the implementation of CE in C&D waste sectors fall in five main domains, namely legal, technical, social, behavioral, and economic aspects. In this context, it was found that policy and governance, permits and specifications, technological limitation, quality and performance, knowledge and information, and, finally, the costs associated with the implementation of CE model at the early stage are the main barriers. In addition to these, from the contractors’ perspective, C&D waste dismantling, segregation, and on-site sorting, transportation, and local recovery processes are the main challenges at the start point for small-scale companies. To address the abovementioned challenges, and also to minimize the ambiguity of resulting outcomes by implementing CE in C&D waste sectors, there is an urgent need to introduce a global framework and a practicable pathway to allow companies to implement such models, regardless of their scale and location. Additionally, in this paper, recommendations on the direction for areas of future studies for a reduction in the environmental impacts have been provided. To structure an effective model approach, the future direction should be more focused on dismantling practices, hazardous material handling, quality control on waste acceptance, and material recovery processes, as well as a incentivization mechanism to promote ecological, economic, and social benefits of the CE for C&D sectors.

## 1. Introduction

**Figure 1:**
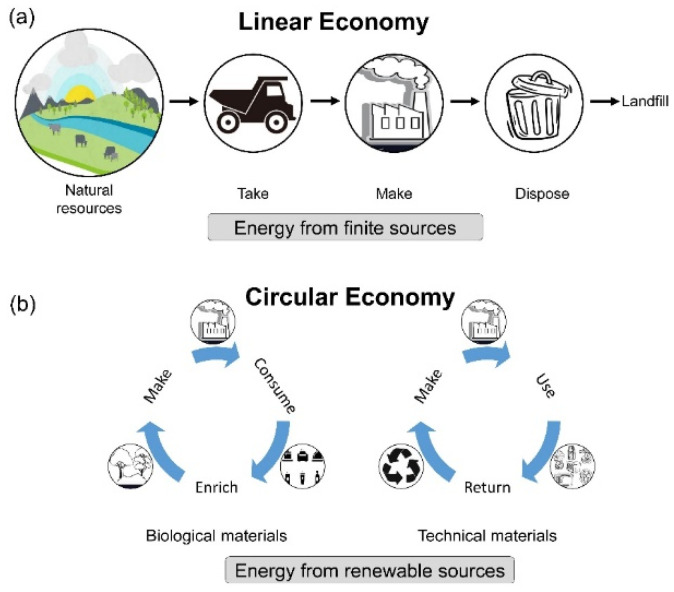
Recycling economy: (**a**) linear economy versus (**b**) circular economy.

Over recent decades, urbanization of the world has exponentially increased coinciding with the human population growth; therefore, the use of more material resources has been amplified. In particular, the construction industry is responsible for utilizing a large portion of natural resources (32%) [1]. Although this value is less than the amount of natural resources used in the 1990s (~40%) [2], it is estimated that more than 75% of waste generated by the construction industry has a residual value and is not currently reused nor recycled. This is due to the lack of integrated waste management framework [1]. Although different definitions have been proposed for Construction and Demolition wastes (C&D wastes), such waste is essentially anything that is produced during the construction, renovation or demolition process that is no longer viable for use. In most circumstances, such waste is discarded to landfill. According to this definition, C&D waste resulting from the construction sector accounts for 30% of total waste produced globally [3], with an estimated average of more than 35% of all C&D waste disposed in landfills annually [4]. Considering the utilization of natural resources, the consumption of considerable amounts of energy and the generation of large quantities of waste through the life cycle of buildings, the construction industry has a significant environmental impact. This is primarily due to the deployment of the linear economy framework which relies on the notion of take, make, and dispose (Figure 1a). In this approach, the raw materials are extracted from natural resources using energy-intensive technology and then processed into fabricating construction materials. Since the majority of construction elements are made in ways that cannot be de-constructed at the end of their lifetime, they are mostly discarded into a landfill or incinerated [5].

At present, C&D waste management poses a significant global challenge due to its negative consequences, including environmental degradation and public health [6]. Such a situation, with contribution to pollution, climate change, and resource depletion, requires an efficient framework to limit said consequences [1,7]. Conventionally, the C&D waste is composed of numerous rejected debris, such as concretes, woods, bricks, glass, steel, etc. [8]. Due to the high volume of C&D wastes that is produced every year, it is becoming vital that construction waste be managed in a sustainable manner. Conventional strategies, such as linear economy, have been identified as inefficient ways to mitigate such negative environmental consequences. In this regard, the term Circular Economy (CE) has evolved as a novel solution to reduce the detrimental effects on the environment and increase economic growth within the construction sector for the practice of sustainable development. Figure 1 illustrates the difference between a linear economy and the circular economy. The CE is comprised of a novel reformative framework that aids in optimizing the consumption of raw materials and ensures the value of materials throughout their lifecycle [9]. In addition to this, CE prevents generation of excess waste, hence preserving natural resources [10,11,12,13]. Essentially, the CE strategy demonstrates that everything that is made can be recycled, reprocessed, or reused. Hossain et al. [10] concluded the main implications of adopting CE for C&D waste as (1) improving the use of sustainable materials which is achievable by integrating the collaborative benefits among all parties involved in the construction project, (2) promoting material efficiency by recycling/reusing the construction wastes, and (3) avoiding the production of unnecessary wastes and consequently disseizing them to landfill. Generally, within the construction industry, the CE aims to add value to the materials that are conventionally discarded into landfill and make them usable for the construction firm or other developments.

To effectively implement CE in a construction firm, various dimensions, such as societal, governmental, economic, behavioral, technological, and environmental aspects, need to be fully elucidated. For example, Ghisellini et al. [14] investigated the costs and benefits of the CE approach specific to the construction and demolition sector. In another study, Lederer et al. [15] employed a material flow analysis to determine how a CE can contribute to the reduction of raw mineral material imports for the construction sector in Vienna city. They found that, by reusing/recycling the construction mineral, the need to import said materials could be reduced by 32%. Although attempts to recycle and/or recover the C&D waste have been made in many cases, there is still limited investigation on practicality of incorporating CE in the modern built environment at a large-scale. Unlike small- and medium-scale construction projects, there are more challenges associated with adapting CE to large-scale applications. Considering that, it is not technically possible to eliminate C&D wastes, the integration of innovative applications, such as BIM (Building Information Modeling), with CE could potentially address the challenges in large-scale built environments. In this regard, a few strategies have been identified which can facilitate the transition toward CE in construction sector. These approaches are (1) utilizing sustainable and durable materials, (2) incorporating design for disassembly, (3) using modular and prefabricated elements, and, finally, (4) development of recovery schemes [10,16]. However, minimal research has been conducted to address the aforementioned approaches up to date.

This review paper will provide an overview on how C&D waste can be reused by implementing a CE strategy from a different perspective. Unlike the linear economy, there are more steps to recover or reuse materials; hence, there are more challenges to overcome. In this study, the challenges, and barriers to implement CE for C&D waste will be discussed based on a new angle of considering the five various construction phases that has mentioned above, along with minimizing environmental impacts and mitigating carbon emissions potential. Proceeding this, the potential use of various materials that have been proven to be effective in construction materials (particularly concrete) will be elaborated. This study helps to further advance the knowledge surrounding construction CE and will also provide a theoretical framework to better promote the use of recycled materials in construction or for other applications.

## 2. Materials and Methods

The strategy used in this study comprised two main stages. In the first stage, a systematic review has been conducted to identify and synthesize research evidence in order to make a generic source of information. This strategy ensures that all relevant, research-based evidence has been collected. For the systematic review of literature, relevant literature was fetched from the largest scientific database, known as Science Direct. To maintain relevancy, the keywords included “construction and demolition”, “waste”, “construction”, “circular economy”, “framework”, “climate change”, “carbon emissions”, etc. The Boolean operators (AND) and (OR) were used separately and in combination for retrieval of relevant publications. Examples of search inputs include: “circular economy” OR “construction and demolition waste” AND “framework”, “construction and demolition” AND “waste”, and “construction waste” OR “demolition waste”, AND “framework”, OR “strategies” OR “management”, etc. The period of search was limited to 2005–2021, with the majority of the publications obtained for this review being from the last five years. This was to ensure that relevancy, novelty, and innovative retrieval of novel ideas and research concepts was maintained. The advantage of this paper to the previously published papers is that the state-of-the-art research in this area is discussed from a new angle of considering the importance of environmental impacts based on C&D waste.

Most retrieved publications were in the English language, as English is a global lingua franca and is widely adopted for communications. Similarly, most of the publications used in this research were peer-reviewed articles published or in press for publication in reputed and well-indexed journals. It should be noted that some of the references also emerged from citations present in the literature. In the second stage, a comparison was made to identify the challenges in implementing construction CE to structure a framework for early adaptation of this effective approach.

## 3. Background Information

### 3.1. Waste

Waste is an inevitable consequence in the production and use of anything, whether it is a by-product in the manufacturing of a material or waste from the demolition of infrastructure. As a society, there is acceptance that waste will always exist; however, due to various factors, such as pollution, resource depletion, reduced landfill space, and climate change, researchers have begun to investigate societies involvement in waste minimization, hence limiting these negative consequences [17,18].

Seadon [19] explains that the ‘mine-build-discard’ viewpoint of society is far from sustainable. The article states that it is historically proven that societies that are not sustainable will eventually fail. Successful societies are ones that understand the importance of their finite resources, use these resources sustainably, and comprehend the complexity of the surrounding ecosystems. Meadows et al. [20] further added that a sustainable society is one that:

- has the ability to develop,
- is advanced both technically and culturally,
- all factors are dynamic especially population and production,
- finite resources are used reasonably and efficiently, and
- is diverse, democratic, and challenging.

**Figure 2:**
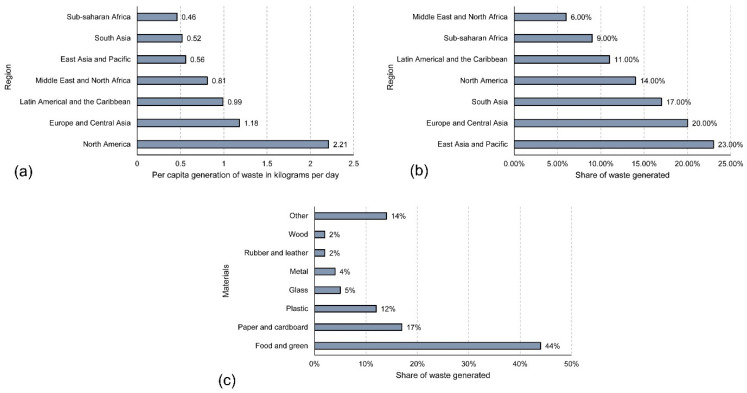
(**a**) Average per capita municipal solid waste generation by region in 2016, (**b**) share of waste generated by region in 2016, and (**c**) global municipal solid waste generation share of materials in 2016 (graphs generated using data from Reference [21]).

The amount of wastes that are generated annually on a global level are at an alarming rate. According to the recent statistics, the generation of municipal solid waste worldwide were recorded to be 2.02 billion metric tons in the year 2016 and is projected to increase to 3.4 billion metric tons by 2050 [21]. From the data in Figure 2a, there is a clear correlation demonstrating that the highest contributors are generally from key countries with high economical contribution into the global market. However, when considering waste-generation worldwide by region, East Asia and the Pacific has the highest, at 23%, with the Middle East and North Africa being the lowest, at 6%, as depicted in Figure 2b [21]. The same statistics revealed the waste breakdown by material type with “food and green” being represented the highest, at 44%, while “wood” and “rubber and leather” materials being least represented, at 2%, as shown in Figure 2c [21].

The projected trend will continue to worsen if the current regime remains; therefore, an increase of 70% of annual global waste production is expected. Addressing this problem requires collective efforts from all members of the international community to ensure the prosperity of our future generation.

Before the COVID-19 pandemic, the New Zealand construction industry was predicted to undergo a growth of 70% by 2029 [22]. This has altered such that it is now predicted to potentially be the largest ‘construction boom’ ever as the government is proposing to increase the amount of horizontal infrastructure projects to ensure job stability for New Zealanders [23]. As such, it can be predicted that waste from construction and demolition (C&D), which contributes to approximately 50% of New Zealand landfill waste, will also increase, potentially putting stress on New Zealand’s waste management infrastructure structure. Recycling of waste materials is one method which can be incorporated into New Zealand’s construction industry to reduce the effects that construction and other industries can have on the environment. One such material that has been proven, through research, to benefit from the implementation of waste is concrete. Tavakoli et al. [24] stated that concrete is, perhaps, the most important construction material used today. Furthermore, he states that, due to the various effects concrete can have on the environment, using waste materials in concrete has the potential to significantly reduce these negative effects.

### 3.2. Sustainability and Circular Economy: Concept and Principles

Sustainability has become a prominent word and/or matter of concern [25] as the world is advancing towards development of urban infrastructure. Consequently, there is a surge in pollution and ill effects on the environment [26]. In any construction project, sustainability is of extreme importance as it brings economic and environmental benefits to the project. As such, a common definition of sustainable development is the assurance that a project accomplishes the needs of today’s generation without compromising the needs of future generations [25]. The principles of sustainability include three entities as its pillars viz. planet, people, and profit. Regarding the planet, the ecology and/or environmental conditions are of extreme importance, while, for people, the development should meet their needs to provide maximum profit within stipulated resources. The principles of sustainable development quest for development to be viable, bearable, and equitable on social, ecological, and economic grounds.

**Figure 3:**
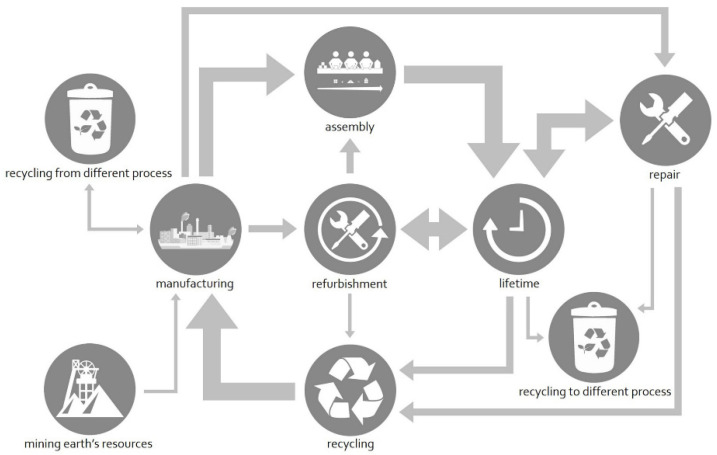
Illustration of material flowchart in the circular economy [25].

The idea of CE also emerged from the need to create awareness regarding environmental degradation resulting from consumption and wastage of raw construction materials. The CE is being considered as a novel solution for the depletion of natural raw materials. Initially, the CE concept emerged out of the 3R’s (Reduce-Reuse-Recycle) principle. This proceeded to become the 4R framework, focusing on reduce-reuse-recycle-recover operations of raw materials [25]. Contrary to the linear economy, in the CE, the raw materials are not disposed of; rather, they are repaired, recycled, and refurbished to be utilized in other processes (Figure 3).

Hence, the principles of a CE include refusing the acquisition of excess raw materials, reforming the design criteria and reducing, reusing, and recycling the waste. Such practices can be deemed efficient in recycling and reducing waste and prevent the current environmental degradation around the globe.

#### 3.2.1. The Hierarchy of Waste Management

**Figure 4:**
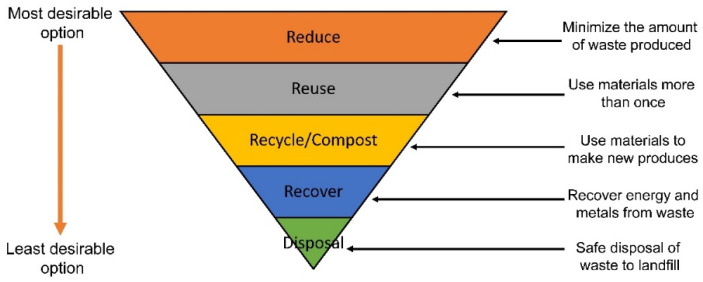
The global waste management hierarchy [29].

Tran [27] argues that waste should not be a residual product but, rather, thought of as a resource that can be continuously reused in an integrated, closed-loop system. BRANZ (Building Research Association New Zealand) states that waste is a good resource and is currently occupying valuable landfill space. Additionally, waste contributes to air and water pollution; therefore, it must be minimized as much as possible [28]. One underlying principle of a CE is the waste hierarchy shown in Figure 4; this is a concept which places the various methods of waste minimization by levels of importance, i.e., the most effective and practical at reducing waste is prioritized.

This globally-used hierarchy begins with the most desirable waste minimization technique; to reduce waste at the source. This simply entails the reduction of excess waste, whether its material packaging or more efficient uses for materials on building sites. The responsibility of the problem falls on companies who create these products or designs buildings or other infrastructure. The second option in the hierarchy is to reuse products/materials once they reach the end of their lifespan. Again, this involves various inputs from different organizations and business, whether its designing for deconstruction on a building site or manufacturing materials which have a long lifespan. The next option is to recycle or compost the materials. Recycling involves finding use for materials which cannot be reused, generally achieved by altering the form of materials to make them desirable for use in other applications or materials. Composting is just recycling that occurs in organic matter, where the materials breakdown and become nutrient rich soil which has many applications. The fourth option is to recover which involves the processing of the waste, in some manner, to produce a valuable outcome. This can include combusting municipal solid waste for energy or recovering precious metals from electronics. The final option, disposal, is the least attractive option on the hierarchy. This is where waste that has no current value is disposed of in a safe manner [30].

#### 3.2.2. Waste Minimization Strategies

The conventional waste management strategies employ the 3R concept of waste management, that is to Reduce, Recycle, and Reuse the construction waste. This involves avoiding the production of waste, reusing the created waste, and recycling of the created waste. Specifically, avoiding of waste, often referred to as waste minimization, entails measures be used for avoiding the generation of waste at the construction site. Alternatively, reusing and recycling are associated with efficient and sustainable utilization of wastes in a viable manner to implement sustainability in construction projects. With these approaches, the volume of waste to be disposed of in landfills is significantly reduced, not only reducing the problems associated with the ecology of the planet but also assisting in conserving the economy due to reduced consumption of physical and non-physical resources required for waste dumping. The construction- and ecology-related professionals recommend that at site reduction and/or avoiding of waste should be prioritized as it is more conceptual and basic to avoid or reduce the waste generation than to formulate widespread frameworks for treatment of wastes. Although, reusing and recycling strategies ensure the waste raw materials are to be used in a beneficial manner, the strategies do not reduce and/or avoid the waste creation at the source. Nevertheless, these approaches aid in reducing the amount of waste to be disposed of or to be treated. It is apparent that recycling or reusing the waste alone cannot be a fully viable option; however, the construction practitioners should take measures to integrate the minimizing and recycling of waste at site to cater the issue of wastes.

While formulating the waste management strategies in any construction projects, the professionals predict potential causes of waste generation from which the site conditions, efficient waste handling methods, and waste management plan should be opted [31]. While designing waste management strategies, the site culture and climate should be taken into the consideration. The strict waste audit and determination of waste index can also be helpful in cutting the waste. Moreover, site security should be carefully implemented, and site inspections should be conducted for check and balance of construction waste generation [32]. An adequate set of planning documents and drawings can also aid in cutting the waste as it eliminates the chance of variation orders [33]. Moreover, the implementation of lean construction strategies and modular prefabricated construction units can also minimize the waste generation [34]. Additionally, management should conduct seminars and meetings to educate the workforce on the importance of waste reduction at site. With a combination of efficient supply chain and improved ordering and storage of materials, the generated waste can be reduced. In the 21st century, a new technology, Building Information Modelling (BIM), has evolved which not only reduces the non-physical wastes (time and cost) but also reduces the physical waste. BIM enables the stakeholders to visualize the number of dimensions of construction project, prior to their commencements, hence avoiding clashes and change orders. Through this technology, the waste generated on site can be reduced by a significant amount [35,36].

## 4. Construction Waste

**Figure 5:**
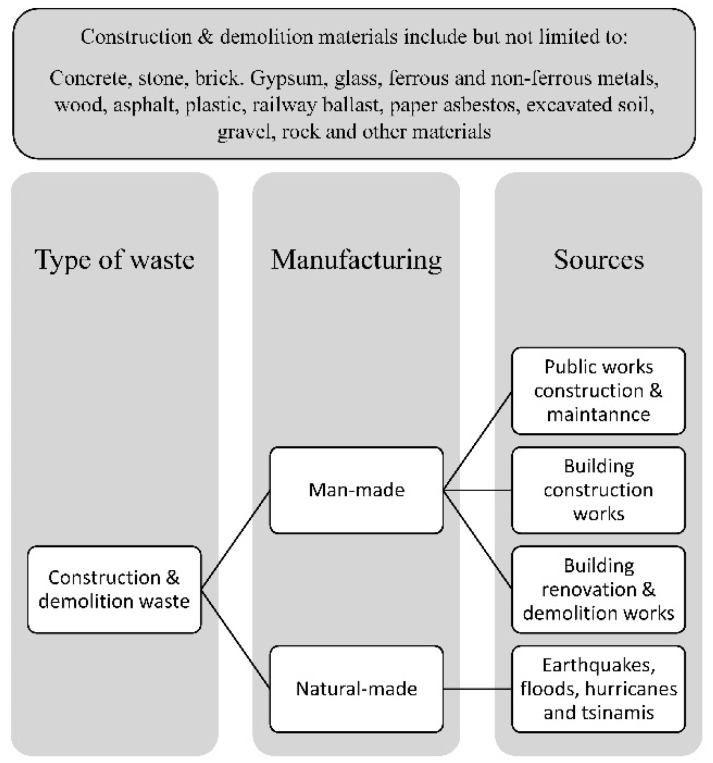
Construction waste types.

Construction waste can be classified on various basis, either on the basis of its source or on its nature. When classified on the basis of its nature, the authors have categorized the waste as physical waste (residual debris) and non-physical waste (time overruns and cost overruns). Figure 5 examines the different sources of construction waste. All construction waste can be divided into two categories, either man-made sources or natural sources, as shown in Figure 5. Upon further examination, the man-made sources can be broken down into the following categories: design, procurement, handling of materials, operation, residual, and other sources [37].

Materials falling under C&D waste are valued items and could be recycled for concrete construction. The major share is in concrete which could be recycled as coarse or fine aggregate [38]. Waste decomposition in New Zealand include wood (38%), plastics (19%), concrete (25%), iron and other metals (6%), organic waste (2%), glass and hazardous materials (2%), and miscellaneous (5%) [39].

**Figure 6:**
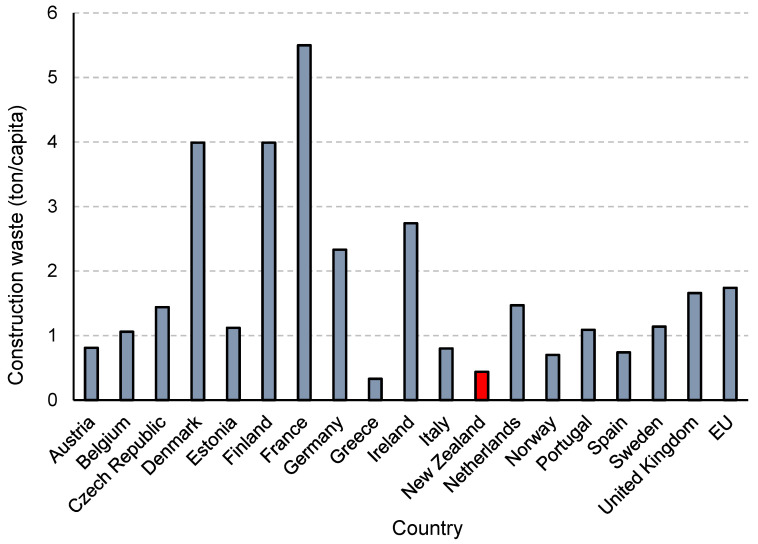
Generation of construction waste (tons/capita) in various countries.

Figure 6 depicts the count of construction waste resulting from construction sector of various European countries, as well as New Zealand, in tons per capita. Evidently, the construction sectors in Denmark, France, Ireland, and Germany are primary contributes of construction waste. Contrarily, Poland, Lithuania, Bulgaria, Greece, Slovakia, Hungary, and New Zealand had the lowest contribution to construction waste generation [40].

C&D waste contributes approximately 50% of New Zealand’s annual waste to landfill; therefore, the construction industry must be significantly involved in the associated waste reduction methods [39]. Whilst the hierarchy of waste minimization places recycling third after reducing and reusing, a 2019 study by the Auckland City Council on the diversion of demolition waste found that recycling would still hold great benefits [41]. The study concentrated on the cost-benefit-analysis of diverting demolition waste from landfill for a new housing project. The study considered two options: option A, where the deconstruction process is completed to a modest level, with a greater focus on partial recovery and recycling of waste materials; and option B, which involves an intensive deconstruction approach with a stronger focus on the two top levels of the hierarchy. Comparison between the two options and the original state or status quo found that, while the developers would just break even, the net benefits to society for both options were significant (A: NPV = $6.97 m, B: NPV = $14.46 m). These benefits include creating jobs for the deconstruction efforts, social benefit from training on the job, economic benefit of construction materials that were reused, reduction of greenhouse gases (GHG’s), unwanted noise traffic and pollutants could be reduced, and skills and experience were gained by workers. While the reduction and reusing intensive option was more beneficial to society, recycling was also found to be beneficial. As recycling is the only option for many materials, it is an important waste minimization method that needs to be investigated further.

## 5. Circular Economy for C&D Waste Management: Feasibility of Waste Minimization

The intrinsic essence of CE lies in reduced disposal of waste into landfills through the utilization of the rejected items in any other viable manner. The CE of construction waste is a 4R solution focusing on Reduce-Reuse-Recycle-Recover operations of raw materials [25]. With greater application of reuse, recycle, and recover operations, the procurement of raw materials becomes slow and/or stagnant, which not only brings economic benefits but also reduces the amount of GHG emissions resulting from procurement and supply chain activities. Moreover, reduced operation of waste is beneficial as it not only reduces the waste but also prevents the consequent negative effects of waste generation on our living environment.

There have been various studies which analyze the economic feasibility of reducing waste. A large portion of such work considers the reduction of C&D waste, as it is the largest contributor to landfills, globally [42]. A cost-benefit-analysis undertaken in Malaysia in 2006 found minimizing C&D waste to be economically feasible with a net profit of 2.5% [43]. This study analyzed the cost and benefits of a construction site in Malaysia minimizing its waste. The study found that there were multiple direct benefits, including purchase cost savings from reusing and recycling materials and selling of scrap metals, waste collection and transport cost savings, and cost savings from landfill charge. Additionally, there were intangible benefits, and these include saving of landfill space, reduced liability for environmental problems or workplace safety, reduced chance of soil and groundwater contamination, and improved public image and environmental concern. The costs that came along with this include direct costs for collection and separation, purchasing of equipment, storage, and transportation. There were also some intangible costs, including risk to workers health and cost of negative externality, i.e., noise and bad smell. These findings are consistent with Auckland City Council’s study which highlights that reducing waste is economically feasible and has a lot of additional benefits which cannot be quantified. One of the key theories mentioned in both pieces of literature was how the increase in wastage levies could incentivize construction companies to incorporate better waste minimization practices.

**Figure 7:**
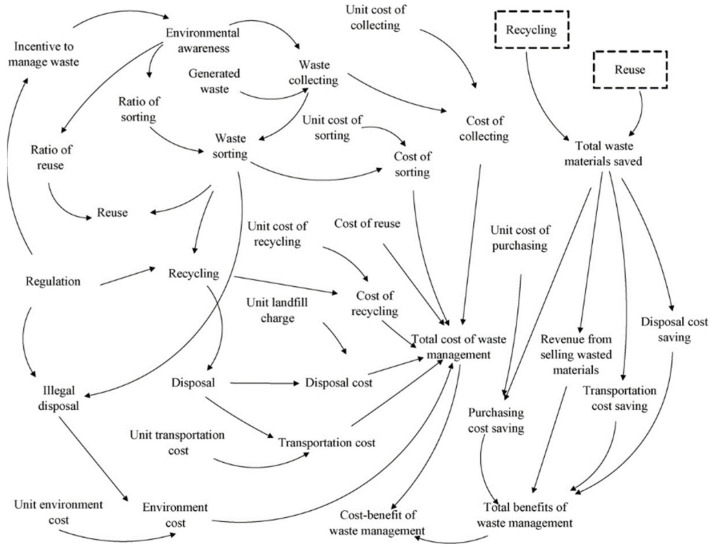
Visual representation of the model created to determine if increased waste levies would increase waste minimization [44].

A group of independent researchers completed cost-benefit-analysis which aimed to test the theory mentioned throughout waste minimization literature [39]. The research was undertaken by creating a complex model of a waste chain which could accurately represent the complexity of waste within society. A visual representation of the variables and their interaction is displayed in Figure 7. This conceptual model emphasizes the complexity of waste reduction and provides reasoning for complications global waste reduction that was previously encountered. The model was tested under four charging schemes, and it was found that a high waste levy would result in higher net benefits for construction companies and society; however, this increase in waste levies provided an incentive for the general public to illegally dump their waste. It was concluded that introducing harsher penalties would be an appropriate measure to combat this problem. Under the four charging schemes, it was found that the cost associated with the first few months after implementing the waste minimization process outweighed the benefits; however, at the 11th month, the benefits began to outweigh the costs. It was also found that the high charging schemes incentivized the contractors to begin waste management earlier, therefore making it more effective. It was found that the lower charging schemes were affected when regulation strengthened, with the net benefits dramatically increasing. It was concluded that waste levies must be higher than 76 yuan/ton ($15.50/ton NZD) to have a worthwhile affect. Not only has waste minimization for the contractor been proven to be economically viable, this result has been proven to occur for recycling companies.

One study investigated the economic viability of using recycled concrete as an aggregate [45]. The cost-benefit analysis was done with both types of practices: current and concrete recycle method for the dumping of waste. The results suggested that, instead of dumping construction waste, particularly concrete, in landfills, the utilization of concrete waste as aggregates can benefit the construction industry [45]. The study found that there was a positive net benefit of $30,916,000 a year, as well as a reduction in resource depletion and energy usage. As such, ecological and economical sustainability can be induced in construction projects. One limitation found was the availability of recycled concrete. It was stated that inconsistent quantities of concrete occurred throughout the study as it is a seasonal waste product, which results in reduced profitability. Additionally, the appropriate materials for recycling are variable as sizes alter, and the location for ‘urban deposits’ is forever changing. Therefore, it is more difficult to recycle concrete compared to the status quo as it is difficult to maintain a predictable revenue stream. This unpredictability is due to uncertainty regarding quantities of material and price fluctuation.

As mentioned earlier, the problem of waste reduction is complex involving many contributing factors. A study completed by Van Tran, in 2017, involved interviewing seven experienced professionals in the construction industry. These interviews were undertaken to gather an understanding on the contributing factors to the poor waste minimization within the industry. Interviewees explained the lack of incentive for construction companies to create better waste minimization programs, stressing that the current waste levy of $10 per ton (2017) was insufficient. The current levy fails to affect a company’s financial bottom line; therefore, it does not encourage them to make changes. It was also agreed upon that the levy fee would need to be increased to $150 per ton, to see drastic action [27]. Such insight is also apparent in the model created by Yuan et al. [44], where a significant increase in waste levies is needed to see a significant increase in change by contractors. This correlation is widely considered to be an effective approach to reducing waste [46,47]. The interviewees also expressed that an incentive would not be adequate in deterring companies from being unstainable, mentioning that penalties should also be implemented on those that continue to not reduce their waste.

In New Zealand, there are currently two incentive schemes to promote sustainable construction; these are the Green Building and Green Star Certification programs. These programs have seen some success in waste reduction, but it is apparent from the vast amount of literature that incentivizing through higher landfill prices indirectly forces construction companies to either reduce their waste or receive reduction to their financial bottom line. While such measures should improve the reduction of waste, there is also the chance that increased landfill prices and penalty schemes will be passed onto the clients. As such, it is important that clients hold their contractors accountable for waste minimization by expressing their need for sustainable construction practices in their project. If the actions mentioned above were taken to assist in the promotion of waste minimization, it would result in more materials being recycled and repurposed. In turn, this could promote financial opportunities, which could result in second-hand markets for materials or niche recycling markets for companies [27]. This will likely increase the feasibility of using waste streams and by-products in construction materials, such as concrete.

## 6. Benefits of Recycling of Construction Materials

The benefits of using recycled materials in construction is driven by the ideology that our natural resources will eventually become scarce if humans continue to mismanage and overuse them, as is occurring currently. Therefore, the benefits will be realized by the three main pillars of sustainability, namely environment, economy, and our society [48]. The following will outline some of the positive impact of using recycled materials in construction.

### 6.1. Environmental Benefits

Maximizing the ability to recycle and reuse construction waste will result in a decreased volume of waste going into landfills, hence prolonging the life of landfill sites for future use. The common use of chemical additives in building materials intensifies the contamination to landfill sites. Some of these toxic substances may find their way into natural waterways and streams through ground water intrusion. Increasing the use of recycled material will consequently reduce the transportation requirements of this waste from the construction site to landfill, therefore decreasing the overall CO_2_ emission contribution.

### 6.2. Economic Benefits

There is an argument that the eradication of landfill use will lead to the loss of employment for those involved in the industry; however, this loss can be counter-balanced by the creation of new opportunities using recycled materials. This is due to the deviation of recycled materials from re-used materials in the sense that, unlike reused materials where they are still in their original form, recycled materials will undergo some form of modification process to enhance the secondary product, while maintaining their physical properties to enable the material or product to serve their purpose in the building. Such processes involve skill sets, hence providing an opportunity for employment. This will contribute to the economy through the provision of such opportunities, yet assisting the cause to reduce the negative impacts on our environment.

### 6.3. Societal Benefits

The continuous population growth will give rise to increased demand of land development. Increased recycling of materials in the construction industry will result in a reduction of land converted to landfills; hence, more quality land would be available for sub-division development to meet housing demands. There are also issues arising from toxic substances from the construction materials that are being disposed of in a landfill that end up in the waterways and natural streams. These uncontrolled scenarios can harm the living organisms found in the surroundings, which can eventually lead to compromising of human health in the community. Additionally, bad odors generated from landfills can be problematic to the nearby community as high winds easily carry them through.

## 7. Recyclable Materials in Construction

With concrete being a highly used construction material that uses a vast amount of finite materials and contributes to CO_2_ production [49,50], it is the perfect candidate for implementing recycled materials to replace cement and aggregate or used as fillers or fibers. The implementation of recycled materials minimizes the waste from waste streams and by-products from manufacturing processes. Numerous studies that highlight the various applications of recycled materials in concrete (Table 1). A review of literature published on these materials was produced in 2018 [24] with some promising results. The study reviewed various waste materials, these included glass, plastic: Polyethylene Terephthalate (PET), tile and ceramics, clay bricks, tires and rubber, metal, concrete waste, agricultural waste, silica flume, fly ash, etc. The study found that waste can be used in concrete, specifically when used in aggregate it can reduce the disposal of large amounts of waste to landfill. When using waste in cement, it reduces the amount of harmful substances in concrete, whilst also being recycled. The specifics of this review are summarized below.

### 7.1. Aggregates Replacement

There are various benefits to the properties of concrete when adding waste as an aggregate. Glass is one material which can increase the properties of concretes. Glass can be crushed into three different forms: Coarse Glass Aggregate (CGA), Fine Glass Aggregate (FGA), and Glass Powder (GP). When glass is mixed with cement, it creates a pozzolanic reaction, which reduces GHGs (CO_2_ and NO_2_) produced in concrete [51]. Additionally, glass has a high thermal conductivity compared to general aggregate; therefore, it can be used on buildings that require thermal stability [52]. Combining both coarse and fine glass together allows for improved water absorption, therefore reducing shrinkage.

PET is a plastic which can be used in concrete, with many believing it benefits the environment [53]. Adding this plastic to concrete can increase its ductility and reduce shrinkage cracks which occur due to moisture changes in the concrete [54]. Another added benefit is that the concrete is lightweight while still maintaining a high quality. Light weight concrete is often used to reduce the dead weight of a structure [55], whilst lowering the workability, density, modulus of elasticity, tensile strength, and slump [56]. Overall, this aggregate is good for lightweight and corrosion resistant concrete.

Tiles, marble, and ceramic are other materials which show improvement to concrete properties when added as the aggregate. Using ceramics as coarse grains (10–20%) increases the concretes compressive strength, while the specific weight decreases without a significant negative affect to water absorption [57,58]. It has also been observed that the mechanical strength of the concrete increases, and maximum water penetration is achieved; however, ceramics are porous and hard; therefore, there is poor water absorption and elasticity. Tiles and ceramics have a low specific weight and good pozzolanic properties. It must be noted that ceramics vary in properties, resulting from their manufacturing process and other variables. This can affect their effectiveness in concrete; therefore, they should be tested prior to use. Fired bricks, which are burnt in a kiln, can be used for sand within concrete. The studies showed that clay bricks as sand could be economical and practical in concrete production. There were no adverse negative effects on the concrete with the exception of corrosion that can occur when used with steel reinforced bars. Overall, there were no added benefits to the concrete properties [24].

Tires and rubber are a waste source that have limited recycling capabilities. The use of rubber in concrete alters this problem as it is beneficial in reducing the stiffness of concrete to protect against fire. An increase in flexural strength was also observed in this study when compared to the control sample. It was also observed that, while the control sample displayed fracture from the brittleness resulting in the sample splitting, the addition of the rubber fiber resulted in deformation, but the sample did not collapse [59]. It was also found that adding silica flume with the cement paste and rubber particles increased compressive strength [60]. Furthermore, adding this fiber improved the freeze-thaw resistance [61]. Overall, rubber seems to be a good additive to aggregate; however, more research must be completed to understand its strength and durability properties.

With 20–30% of agricultural production ending as waste, it is important to optimize the amount recycled. Current research for agricultural waste used in concrete has utilized the shells of almonds and coconuts. A study tested the use of almond shells as a coarse aggregate, which produced average slump, increased air content, and lower air density compared to ordinary concrete [62]. Following this, another study produced a lightweight good quality concrete using coconut shells [63]. It was found that coarse grain aggregate had a lower weight and the same mechanical properties as that of normal coarse grain aggregate. It also demonstrated decent quality and flexural behavior identical to the ordinary sample. Recently, it was found that the compressive strength of concrete with coconut shell can be reduced by 22%, which may be mitigated through the reduction of the water-cement-ratio [64].

**Table 1:** Summary of various benefits of different wastes which can be incorporated as aggregates in concrete.

Material	Benefits	Refs.
Glass	Pozzolanic in nature, high thermal conductivity, reduced shrinkage, improved water absorption, reduced ecological emissions.	[51,52]
Plastics	Increased ductility, reduced shrinkage cracks, lightweight concrete.	[53,54,55,56]
Ceramics	Enhanced strength, required water absorption, low specific weight, and high pozzolanic nature.	[58]
Rubber	Protection against high temperatures and increase in strength.	[59,61,65]
Concrete	Pozzolanic in nature, high thermal conductivity, reduced shrinkage, improved water absorption, reduced ecological emissions.	[66,67]
Coir & Almond Wastes	Increased air content, improved mechanical strength and lower air density.	[52,68]

### 7.2. Supplementary Cementitious Materials (SCMs)

Concrete waste research began as far back as World War II, making it the earliest recycled material in concrete. The list of potential SCMs to be used as a replacement for cement or aggregates have been listed in Table 2. It was found that adding fly ash to the mix helped to prevent shrinkage that was due to the addition of the concrete waste [66]. Another study found that the use of clay brick powder as cement compensated for the decrease in compressive strength due to the waste aggregate [67]. The research indicates concrete waste is a viable recycle material to be used as an aggregate; however, caution should be taken when using it as different projects require different concrete properties, and the specific amount of waste can greatly affect the concrete performance.

In the production of metal, 17% of the material becomes a by-product known as slag [24]. It was found that using this by-product to substitute the coarse grain resulted in high shear modulus and chemical stability in alkaline and acidic solutions [68]. A study found that mixing slag in high performance concrete produced concrete which was higher in water absorption, tensile strength, and compression strength [69]. Another study found that, while the slump increased, as expected, the density and bending strength also increased [51,70]. Alternative studies have shown that there is the potential to achieve ultra-strong concrete at around 150 MPa. Overall, the hardness of steel furnace slag relative to traditional aggregates is much higher, therefore increasing the flexural and compressive strength; however, it is important to note that adding slag to concrete increases its weight.

Silica fume is a by-product of the production of silica metal which can improve Portland cement production properties due to its ‘super pozzolanic’ properties. One study found that substituting 10–15% of the cement with silica flume increased the strength properties in the early drying stages [71]. Alternatively, another study suggests that silica flume could have harmful effects on the durability of concrete [72]. This result is not desirable; therefore, silica fume should only be used as per requirements and design criterion as it has some negative consequences. In another study, Zhang et al. [73] investigated the effects of nano silica particles on the impact resistance, mechanical properties and durability performance of concrete supplemented with coal fly ash. The authors added various percentage of nano silica (1–5% of the binder weight), and it was found that the modified concrete with nano silica has a better mechanical properties, along with a better freezing-thawing resistance performance. More specifically, the addition of nano silica resulted an increase in compressive, flexural, and splitting tensile strengths of the samples by 15.5%, 27.3%, and 19%, respectively. The literature also shows that the addition of nano silica can be beneficial for basalt fiber-modified recycled aggregate concrete [74]. This combination could be useful when using recycle aggregate as the inclusion of basalt fiber can reduce the generation and propagation of primary microcracks in recycled aggregate concrete, as well as mortar. In this regard, nano silica acts as a filler to fill the microcracks and also promoting the cement hydration. Another investigation shows that the addition of certain nano silica can help geopolymerization of the mortar; however, further increasing the nano silica content than its critical ratio can negatively impact the mechanical properties [75].

When rice husk is burned, its pozzolanic properties increase, making it a very desirable waste material to be added to concrete. One study found that adding rice husk to a high-performance concrete with micro silica resulted in hydration of the cement, hence reducing the porosity of the cement [76]. Additionally, the compressive strength and water absorption were observed to improve. Alongside this, it was discovered that resistance to a chloride attack was approved in addition to compressive strength and other mechanical properties [77]. It should be noted that, for countries with limited aggregate production facilities, rice husk can be beneficial as an addition to concrete as it can be used in high strength concrete or repairing mortars. The use of rice husk in concrete products as a cement additive is practical; however, the waste needs to be used at an optimal level to achieve the desired properties.

Coal fueled power plants produce a by-product from the burning of coal known as fly ash [78]. It was found that the use of fly ash, replacing 40–60% of the cement, results in an increase in the compressive strength in 28 days compared with ordinary cement [79]. Additionally, class F fly ash with good pozzolanic properties results in good mechanical properties, durability, and low chloride permeability [80]. The literature has shown that the addition of fly ash can increase mechanical properties of concrete, and, unlike concrete waste and brick, this material will not corrode the steel reinforcement. A study showed that, when 10% of glass powder is used in a cement mixture, it outperforms fly ash on compression strength in the early curing stages; however, at 90 days, fly ash produces a concrete with a higher compression strength and water absorption [81]. To reduce an Alkali–Silica Reaction (ASR), which is detrimental to concrete performance, it is important to add silica to concrete as an admixture.

Early studies on the chemical properties of the ceramic tile found that it had pozzolanic properties [82]. A study concluded that the use of clay brick waste in cement could be replaced, achieving 91% of the strength of ordinary concrete [83]. Additionally, the replacement cement reduced permeability of concrete and increased efficiency. Alternatively, tile powder can be used alongside silica flume to produce a concrete which has similar properties to the controlled sample [24].

**Table 2:** Summary of various benefits of different wastes which can be incorporated as supplementary cementitious materials (SCMs) in concrete.

Material	Benefits	Refs.
Metal Slag	High shear modulus, chemical stability, high strength.	[51,69]
Silica Fume	Pozzolanic nature, increased strength.	[71,72]
Rice Husk Ash	Enhanced compressive strength and improved water absorption.	[76,77]
Coal Ash/Fly Ash	Pozzolanic nature, good durability, low permeability, increased mechanical strength, reduced the alkali–silica reaction.	[78]
Ceramic Wastes	Increased strength, reduced permeability of concrete and increased efficiency.	[84]

## 8. Current Regulations, Barriers and Challenges in CE for C&D waste

In an ideal world, all construction waste would be recycled and reused; however, there are many barriers which prevent this from happening. A case study in Australia [85] found that the six main barriers to C&D being recycled are as follows.

### 8.1. Policy and Governance

One of the biggest barriers to construction waste reuse in New Zealand is the lack of policy related incentive for companies. Current legislation for construction waste in New Zealand includes the Building Act (2004) and the Waste Management Act (2008). The Building Act (2004) implements the sustainability principles of the Ministry of Business, such as “the efficient and sustainable use of materials” and “the reduction of waste during the construction process” [86]. The Waste Management Act (2008) encourages the reduction of waste by implementing a $10 per ton levy for all waste products sent to landfill. The levy was used to incentivize waste reduction, whilst simultaneously generating revenue to develop new technology and practices in the industry. It should be noted that the imposed levy did little to change to landfill patterns, with construction waste continuing to rise.

### 8.2. Quality and Performance

Another barrier to the recycling of C&D waste is the need for it to be in quality condition. In order to make sure materials are of a high quality, they need to be manually sorted. Manual separation requires both time and money, hence increasing the pressure on an already strained system. Separation of materials is particularly important regarding hazardous materials, exemplary of this is timber. When regarding timber separation, it is important that contaminated wood be separated from non-contaminated wood which may be achieved on site or at transfer stations. If the material is sorted on site, then the associated cost is in terms of labor required to separate the material in addition to storage costs. If the material is not properly sorted, then it is unable to be recycled; thus, good separation practices need to be adopted for large amounts of material to be recycled.

### 8.3. Information

There is a lack of information within industry of the importance of recycling and the potential associated benefits. The construction industry is very dynamic, yet the practitioners have yet to understand the essence and importance of recycling the materials to avoid the waste. The respective organizations need to inform the workforce of the benefits of recycling materials for construction activities, using conducted case studies as examples [10].

### 8.4. Cost/Capital

In any construction project, the associated cost is of extreme importance as it is considered a major performance indicator and driver for success of the project. Unfortunately, in New Zealand, it is currently more expensive to recycle a material than it is to send it to the landfill [41]. Table 3 shows the current cost to recycle material in New Zealand.

### 8.5. Perception and Culture

Often, the value within C&D material is not fully realized, with many in the industry not considering it is as a potential resource. It is apparent that the majority of construction professionals consider waste as merely a waste and not a potential resource. Globally, there is increased focus on recyclable and renewable technologies in order to meet the sustainable development goals; therefore, it should be mandatory that construction practitioners update their perceptions and shift their focus from conventional methods to newer technologies [87].

### 8.6. Knowledge, Education and Lack of Technology

There are many companies and workers within the construction industry that do not have access to education on the circular economy. Education is a key factor to inducing change, with people traditioned to the norm unless educated otherwise. It is the sole responsibility of construction professionals to learn the importance of recycling of materials and, subsequently, encourage their workforce to do the same. Nevertheless, governments and regulatory authorities are also responsible in conducting such educational seminars, meetings, workshops, etc., to update the knowledge and education of construction sector workforce. Generally, the absence of technology required for waste recovery and recycling results in contaminated, low-quality products. As such, the cost of acquisition of recycled materials is high, while the performance of the materials is relatively low and not up to the desired standards [87].

### 8.7. Permits and Specifications

Specifications and standard requirements put negative influence on applications of recycled materials. Moreover, there are numerous causes that result in permits for the utilization of recycled materials in certain projects to not be granted. With such barriers, the associated market becomes uncertain regarding the production and availability of recycled materials.

## 9. C&D Wastes Effects on Greenhouse Gases Emissions

Greenhouse gases (GHG) emission was responsible for global warming and climate change in the past few decades. The major GHGs responsible for global warming are water vapor (H_2_O), methane (CH_4_), ozone (O_3_), nitrous oxide (N_2_O), chlorofluorocarbons (CFCs), and, most importantly, carbon dioxide (CO_2_) [88]. Cement is one of the most important key components in construction works where concrete and other construction material derives from cement. In addition, cement and concrete significantly contributes to C&D waste sent to landfill and which creates extra GHG emissions.

The cement industry alone contributes to about 7% of global CO_2_ emissions due to the nature of the cement production process [89]. The majority of CO_2_ emission in cement production is due to thermal calcination of calcium carbonate (CaCO_3_) stone known as limestone in a cement kiln where quick lime (CaO) and a large quantity of carbon dioxide are produced.

(1) $${\mathrm{CaCO}}_{3}+\mathrm{Heat}\to \mathrm{CaO}+{\mathrm{CO}}_{2}.$$

Thermal decomposition of calcium carbonate to produce one ton of clinker approximately produces 0.51 t_CO2_; in addition, to produce the required heat of calcination process, typically, a significant amount of fossil fuels (including fuel oil, natural gas, coal, etc.) is burnt, which produces CO_2_ and other GHGs. Therefore, to produce one ton of Portland cement, approximately 0.8 t_CO2-eq_ is emitted [90]. On the other hand, the world’s cement production is increasing by 2.5% annually due to rapid growth in urbanization and industrialization of developing countries [91]. Therefore, considering the high environmental impacts and GHG emissions potential of both cement and construction industries, it is essential to take steps and develop mitigation strategies to control and reduce CO_2_ emissions in the sector. In the past few years, several mitigation strategies have been implemented to reduce the negative impact of the construction industry on climate change, in general, which are (1) increasing energy efficiency in both cement and construction industries; and (2) using alternative fuels (e.g., biofuels, municipal wastes, scrap tiers [92] in cement kiln); clinker substitution/blended cement; reuse of C&D waste using circular economy concept [93].

The C&D waste end life disposal methods, including landfill and incineration, significantly contribute to GHG emissions, where C&D waste accounts for 46% of total waste in the EU [94], 40% total municipal waste in China [12], and 20% of total solid waste in Japan [95]. According to Andrade et al.’s [96] estimation, global C&D waste will be increased from 12.7 billion metric tons in 2018 to 27 billion metric tons by 2050, which indicated an urgent action is needed to restrict C&D waste CO_2_ emissions. Xu et al. [97] developed a new Building Information Modeling software tool to quantify the amount of CO_2_ emissions for the C&D waste end of life disposal process in China. They calculated total emissions based on transportation, recycling, and landfill emissions, which shows the potential for further emissions reduction by implementing the circular economy concept in the construction sector, as shown in Table 4.

Kaliyavaradhan and Ling [98] studied the potential of carbon dioxide sequestration through C&D waste by the mean of recycled concrete aggregate. They concluded that CO_2_ sequestration through C&D waste is a promising solution to reduce GHG emissions and to achieve a realistic balance of the CO_2_ cycle in both cement and construction industries. Corsten et al. [99] showed the application of sustainable waste management significantly reduced GHG emissions and energy use in the Netherlands. In their research, three main waste streams were defined as household waste, bulky household waste, and C&D waste. Their results concluded that high-quality recycling can reduce emissions by 2.3 Mt_CO2-eq_/y compared to the reference situation in the country in 2008. The study on the environmental and economic impact of C&D waste disposal using system dynamics in Egypt showed that recycling and reuse of such wastes significantly reduce energy use, global warming potential, along with GHG emissions reduction, and conserve the landfills’ space [100]. Recycling C&D waste can approximately reduce the need for primary raw material to around 12.3 million tons by 2024. It also reduces the CO_2_ mitigation cost by $16,161.35 billion over a 20-year period of conducted study. In addition, Islam et al. [101] conducted research on C&D waste of Bangladesh as an example of a developing country that shows, in 2016, in Dhaka city, approximately 1.28 million tons of C&D waste were generated and sent into landfill or unauthorized places. Their study showed that recycling these wastes can contribute to the national economy of around $45 million and, likewise with a similar proportion, can contribute to emissions reduction.

## 10. Frameworks and Model Approaches for CE for C&D Waste

The review of literature has revealed that there is limited research on integration of circular economy and construction industry in Australasia, particularly in New Zealand. If CE were adopted in the construction sector, the environmental and economic issues can be reduced. In a model formulated for integrating CE into the construction sector, it was claimed that such integration can be achieved successfully in three layers or three stages via micro, meso, and macro. The authors perceived that micro stage of CE need to focus on eco-friendlier design and cleaner processes, and meso for frameworks accelerating waste trading systems. The most important, macro, needs to deal with the 3R principles among collaborative industries comprising numerous stakeholders [102,103]. Fewer countries, such as the UK and the Netherlands, have integrated CE in construction firms. For instance, the UK has adopted an approach called Resource Efficient Construction, which not only reduces the waste but also cuts the GHG emissions resulting from construction activities. The approach aids the construction practitioners in redesign of the debris as a resource, development of recycled materials, as well as boosting up the process of reuse and recycle. Taking this framework, Ellen MacArthur Foundation has developed a six-stage framework called ReSOLVE, with the following points:

- Regenerate: Encouraging to move the focus from traditional to renewable technologies and prevent the destruction of ecosystem.
- Share: Driving towards increasing the lifespan via efficient maintenance schemes and sharing the recyclable and reusable resources and assets.
- Optimize: Enhancing the efficacy of recycled goods by cutting unwanted wastes via efficient and green supply chain.
- Loop: Providing the required technology to recreate and recycle the wastes.
- Virtualize: Dematerializing in both direct and indirect way.
- Exchange: Encouraging and enhancing the adoption of innovative construction materials and newer techniques.

A questionnaire-based pilot survey in Denmark has revealed that, among the ReSOLVE framework, there are strong chances for the construction process to undergo share, optimize, and loop stages of said framework [104]. In the Netherlands, an organization with the name International Management Search Association (IMSA) is very dynamic in integrating the CE in construction activities [105]. The framework proposed determined that an efficient construction waste management plan can be formulated by solving the issues of increasing waste, negative repercussions on ecology of planet, illegal dumping of waste, and absence of support from the top tier of construction organizations.

In another investigation, Esa et al. [106] developed a framework for CE integration in construction firms, focusing on the involvement of the 3Cs (Contractor, Consultant, and Client) in the 3R operations of construction waste in the five stages of the project lifecycle, i.e., planning, designing, procurement, construction, and demolition. On a micro level, the authors urged the adoption of “Industrialized Building System (IBS)” for efficient and sustainable facility management. At a meso level, it was suggested that the regulations concerning the construction sector should be brought in to action for reduction of waste and encouragement of sustainable development. As the complete eradication of waste is not feasible, at the macro level, the authorities should manage the C&D waste through efficient surveillance mechanism on the workforce. This framework and the roles of various stakeholders at various stages, including planning, designing, procurement, construction, and demolition of project lifecycle can be found in the model the authors proposed [104]. The numerous waste reduction strategies discussed above, at micro, meso, and macro levels, are discussed in line with the 3R operations of efficient C&D waste management.

**Figure 8:**
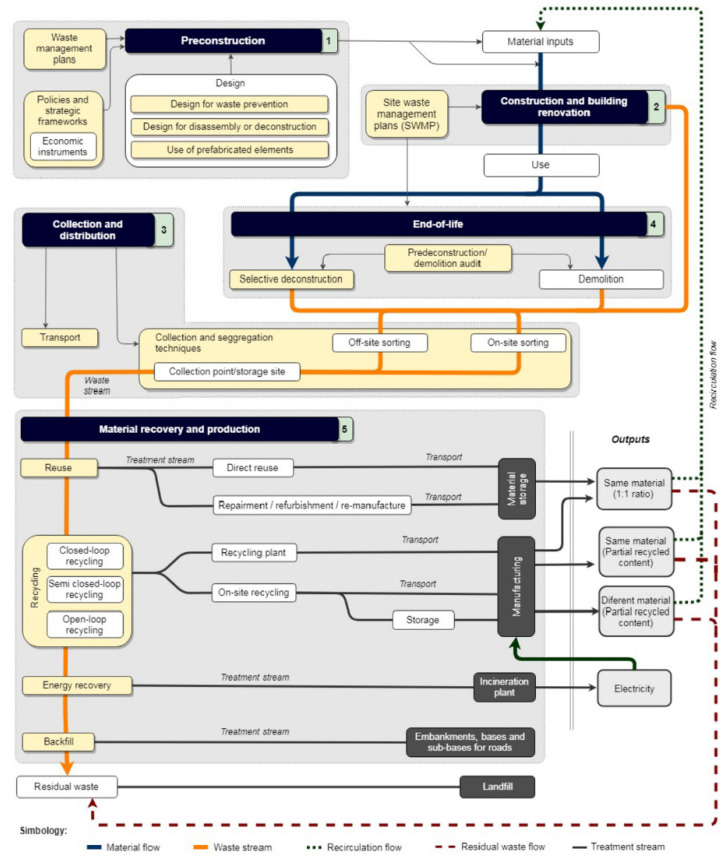
Framework for circular economy adoption in construction [107].

Another integrative framework for adoption of CE in C&D waste management was proposed by Ruiz et al. [107], which focused on formulating the strategies for C&D waste management in five lifecycle stages, i.e., preconstruction, construction and renovation, collection and distribution, demolition and material recovery, and production. Overall, fourteen strategies were proposed as mentioned against every stage and are detailed in Figure 8:

- Preconstruction: enforcement of government regulations, taxation on acquisition of raw materials, employment of economic instruments, and prioritization of waste recover options.
- Construction and Renovation: selective destruction, efficient waste management plan.
- Collection and Distribution: Collection and segregation practices, on-site sorting, efficient distribution of resources, transportation and recirculation of recyclable and recycled materials.
- Demolition (End of Life): preference to selective deconstruction over traditional demolition, waste audits and material recovery, etc.
- Material Recovery and Production: Reuse, recycle, backfilling and recovery of material and/or energy, waste treatment processes, ecological and economic aspects of waste recovery.

## 11. Scientific Reuse Perspectives

Although there are numerous benefits in transitioning to circular economy in C&D sector, the scientific reuse of such wastes needs to be investigated. The recovery process should be in a way to result in an acceptable quality prescribed by specifications and be economically feasible to encourage contractors to recycle and also use the recycled C&D waste materials. In this regard, the chemical composition as well as physical, mechanical and durability performance of recycled waste materials need to be examined. In addition to the properties of recycled materials, from an environmental point of view, the sustainability and life cycle of recycled materials.. Recycling is, by nature, an energy-consuming process; however, in most cases, when considering the social and environmental benefits, the process outweighs discarding C&D materials in landfills; therefore, the recycling becomes feasible. For example, in C&D waste recovery, energy for mining, quarrying, and transportation has already been expended in the first life of recycled materials. This can also reduce the emission and other environmental impacts, such as natural resource depletion and dust/airborne spreads. However, to gain a more in-depth insight, the life cycle analysis can be deployed to assess the contribution of CE to sustainability. In this context, Butera et al. [108] explored the life cycle of C&D waste management by considering both toxic and non-toxic environmental impacts. The authors found that transportation is the most contributing factor (60–95%) for non-toxic impacts. Interestingly, landfilling minerals had lower impacts than utilization. This is mainly due to a lower levels of leachate per ton of C&D waste materials that reaches ground water resources over a 100-year period. However, leaching oxyanions was found to be critical aspect, and, in this domain, Cr immobilization is soils was found to be pivotal. Overall, the findings show that leaching emissions had a significant influence on toxicity impacts in comparison with the production of the same materials. As expected, CO_2_ uptake due to transportation (on-site and off-site) was found to be 15% from the life cycle analysis.

## 12. Conclusions, Limitations, and Future Directions

Annually, a large portion of waste generated from C&D ends up in landfill, which has led to sever environmental and social problems. The amount of C&D waste disposed is continuing to grow at an alarming rate, resulting in negative consequences. Currently, there are a few model practices in place to recover or reuse a small portion of C&D waste; however, to be deployed globally, numerous challenges/barriers must be overcome. In this regard, the literature demonstrates that the challenges can be classified in five main categories, including legal, technical, social, behavioral, and economic barriers. Within the primary domains, policy and regulations, permits and specifications, technological limitation, quality and performance, knowledge and information, and the costs associated with the implementation of CE model at the early stage are the challenges to be addressed. In addition, it was found that the scale of challenges varies depending on the scale of the projects and also varies from countries to countries. Therefore, a general framework and practicable pathway is needed for a successful transition from linear economy to a circular economy in C&D management sector. However, to be able to propose a framework, there is an urgent need to explore and classify the monetary and social benefits of recycling C&D waste and identify the key associated challenges. To this end, numerous integrative models and frameworks from various regions, such as the UK, the Netherlands, Malaysia, Denmark, etc., were reviewed and critically discussed. This review paper investigates the challenges/barriers in implementing CE in C&D waste and explores the feasibility and benefits of recycling construction waste materials. In this regard, the ecological, economical, and strength and/or durability associated findings from previous literature were uncovered. It was found that the recycling of waste materials in construction has positive influences on environmental, economic, and durability characteristics of the construction activities. As such, the green and sustainably built environment will guarantee good public health and can help government and stakeholders to meet sustainability goals. Additionally, the resources to be spent on formulating the waste management plans and workforce required for their implementation can also be abridged; however, there are numerous barricades faced by the construction sector preventing the recycling of C&D waste materials in construction activities. The identified bottlenecks involved strict quality assurance systems, market uncertainty about availability of waste materials, knowledge and negative perceptions, high cost of material recovery related technologies, etc.

The review of available extensive literature has led to the following recommendations for efficient integration of CE concepts in the construction sector for sustainable development:

- The waste obtained from C&D activities should be efficiently dealt with and handled such that its quality is not impaired; therefore, its utilization as aggregates or cementitious resource should remain feasible.
- Selective demolition should be practiced for hazardous materials, such as tubes, asbestos, etc. The handling should be efficient so that mixing does not occur, which can cause contamination of recyclable materials.
- On-site sorting should be practiced such that mixing of waste may be avoided. The waste should be classified on basis of nature and possible economic benefits.
- Efficient quality control systems should be enforced with proper check and balance on method of material recovery, waste acceptance criterion, material properties, and pros and cons of material utilization in construction activities.
- As the concept of CE in the construction sector is not mature, the local and central governments should come forward and play their part in enlightening the organizations regarding the ecological, economic, and social benefits of the CE approach.

## Figures and Tables

**Table 3:** Cost of recycling the materials in New Zealand.

Costs	$/tonne
Sorting-wood	40–126
Chipping-wood	20
Sorting-concrete	7
Preparation-concrete	4
Crushing-concrete	8

**Table 4:** CO_2_ emissions in C&D waste in China [97].

Construction Material Type	Transportation Emissions (t_CO2-eq_)	Recycling Emissions (t_CO2-eq_)	Landfill Emissions (t_CO2-eq_)	Total Emissions (t_CO2-eq_)
Soil	55.3	41.2	180.8	222
Concrete	280.9	1274.6	5965	7239.6
Brick	66.7	24.2	326.8	351
Cement	45.1	16.4	147.4	163.8
Lime	0.055	0.02	0.18	0.2
Mortar	108.4	39.3	353.9	393.2
Steel	17.8	170.8	87	257.8
Ceramic tile	12.6	5.7	37.1	42.8
Paint	0.0034	0.02	1.26	1.28
Polymer coating	0.2	1.1	74.9	76
Plastic	0.25	1.5	0.82	2.32
Wood	0.19	1.7	1.53	3.23
Paper	0.0021	0.06	0.79	0.85
Asphalt	7.6	41.5	2861.2	2902.7
Plaster	0.82	0.88	2.69	3.57
Total	596	1618.2	10,042.2	11,660.35

## Data Availability

Not applicable.
